# Surgical treatment of fourth branchial apparatus anomalies: a case series study

**DOI:** 10.1186/s40463-020-00477-8

**Published:** 2020-11-16

**Authors:** Wan-Xin Li, Yanbo Dong, Aobo Zhang, Jun Tian, Cheng Lu, Jean Pierre Jeannon, Liangfa Liu

**Affiliations:** 1 Department of Otolaryngology Head and Neck Surgery, Beijing Friendship Hospital, Capital Medical University, 95th Yong’an Road, Xicheng District, Beijing, 100050 China; 2 Surgical Oncology, Guy’s & St Thomas NHS Hospital, Kings College London, London, UK

**Keywords:** Fourth branchial apparatus anomalies, Complete surgical excision, Direct laryngoscope, Superior laryngeal nerve, Pyriform fossa apex

## Abstract

**Background:**

Fourth branchial apparatus anomalies, are rare clinical entities, and present as complex cysts, sinuses and fistulae in the neck that can be difficult to manage.

**Methods:**

This is a retrospective review of a series of consecutive patients with fourth branchial apparatus anomalies treated at Department of Otolaryngology Head and Neck Surgery, Beijing Friendship Hospital, Capital Medical University, from Apr 2014 to Nov 2019.

**Results:**

Ten patients with fourth branchial apparatus anomalies were identified, including 8 patients with fourth branchial fistula, and 2 patients with fourth branchial pouch sinus. There were 6 female patients and 4 male patients. Their age was from 6 years old to 39 years old (average age 20.4 years old, median age was 21 years old). All 8 fistulae were on the left side, while 2 pouch sinuses were both on the right side. Pre-operative examination with fiberoptic laryngoscope, barium swallow X-ray, CT or MRI identified internal orifice at pyriform fossa apex in 8 (80%) patients. All patients underwent challenging surgical resection by the senior author. Intra-operative direct laryngoscope confirmed or identified internal orifice in 9 (90%) patients. The tracts were all followed to the vicinity of inferior cornu of the thyroid cartilage and the cricothyroid space. Complete resection of cervical lesions and their attachment to hypopharynx were achieved in 9 cases. No complication occurred. One recurrence was detected, in the only patient whose internal orifice could not be located pre- or intra-operatively, and the hypopharyngeal attachment could not be removed.

**Conclusions:**

Direct laryngoscopy under general anesthesia is a reliable method of diagnosis for the fourth branchial apparatus anomalies. Complete surgical removal of fourth branchial apparatus anomalies, including their hypopharyngeal attachment, is the treatment of choice, and the key to prevent recurrence.

## Background

Branchial anomalies are congenital conditions which results as a consequence of aberrant embryonic development of the branchial apparatus. A spectrum of conditions can result due to failure of the coordinated branchial alignment which include branchial cysts, fistulae, and sinuses. These may occur in any age, but the first and second decades of life are the most common [1]. Anomalies of the second branchial apparatus are the most commonly seen branchial defects, accounting for between 75 and 92% of cases depending on the case series. Anomalies from the third and fourth branchial apparatus are less commonly encountered, and seen 2 and 1% of the time respectively [2, 3].

Branchial anomalies (including cyst, sinus, and fistula) result from abnormal persistence of branchial apparatus remnants. A cyst is an epithelial-lined structure without an external opening. A sinus is a blind tract with an opening either externally through the skin (representing persistence of a branchial groove) or internally into the foregut (representing persistence of a branchial pouch) [4]. Branchial cleft sinus opens to the skin only, while branchial pouch sinus opens to the pharynx only [5]. A fistula is a tract that communicates between the skin externally and the foregut internally (representing persistence of a branchial groove with its corresponding pouch, with no branchial membrane between them) [4]. Hence, we believe the term incomplete branchial fistula is a misnomer, and should be called branchial pouch/cleft sinus.

Fourth branchial apparatus anomalies tend to occur predominantly on the left side [6], with an external orifice on the lower neck in the line of the anterior border of the sternocleidomastoid muscle, and an internal orifice at the pyriform fossa apex (PFA). They can present as repeated episodes of neck swelling, abscess formation, and even suppurative thyroiditis [7]. Some authors advocate a conservative approach utilizing endoscopic cauterization of the internal opening, including: chemo-cauterization with trichloroacetic acid [8] or silver nitrate [9], electro-cauterization [10], or laser diode cauterization [11], of the fistulae. However, complete surgical excision remains the definitive treatment of choice [7, 12, 13].

Diagnosis of fourth branchial apparatus anomalies can be difficult and may only result after frequent presentations to medical care. In the neonate, these anomalies can present as problems with feeding and respiratory symptoms. Rarely rapid enlargement of the anomaly can result as the infant swallows saliva, formula, or milk, leading to tracheal compression and respiratory distress [14].

Clinical confirmation can be made by endoscopic visualization of an opening at the PFA [15]. If an internal opening can’t be visualized, differentiation from third branchial apparatus anomalies can only be achieved by intra-operative dissection. Since third and fourth branchial fistulae both originate from the pyriform fossa, they are collectively referred to as pyriform fossa fistula [12]. The relationship of the tract to recurrent laryngeal nerve (RLN) and superior laryngeal nerve (SLN) is the key in differentiating between the two entities. If the tract courses cephalad to the RLN and caudad to the SLN, and reaches hypopharynx around the inferior cornu of the thyroid cartilage, this indicates a fourth pouch origin [5, 15]. A tract that exits from the rostral aspect of the pyriform fossa, pierces the thyrohyoid membrane cranial to the SLN and inferior constrictor, identifies a third pouch origin [16].

Here, we present our experience in the management of 10 cases of fourth branchial apparatus anomalies, over a 5-year period in our tertiary academic institution.

## Material & Methods

This is a retrospective study of patients with fourth branchial apparatus anomalies (including fourth branchial fistula and fourth branchial pouch sinus), treated at Department of Otolaryngology Head & Neck Surgery, Beijing Friendship Hospital, Capital Medical University, from April 2014 to Nov 2019. The clinical and demographic data was retrieved from the case notes. Median follow up was 4 years.

### General material

Ten patients with fourth branchial apparatus anomalies were identified, including 8 patients with fourth branchial fistula (Patient No. 1 and 3–9), and 2 patients with fourth branchial pouch sinus (Patient No. 2 and 10). There were 6 female patients and 4 male patients. Their age was from 6 years old to 39 years old (average age 20.4 years old, median age was 21 years old). All 8 fistulae were on the left side, while 2 pouch sinuses were both on the right side, as shown in Table 1.

**Table 1:** Demographic data, preoperative examination and intraoperative confirmation of internal orifice

Patient No.	Age	Gender	Side	Drainage and previous attempts of surgical excision	IO at PFA on pre-operative fiberoptic laryngoscope	Outflow of barium from PFA on pre-operative X-ray	IO confirmation by Intra-operative direct laryngoscope
1	12	Male	Left	4 times	Yes	Yes	Yes
2	7	Female	Right	no	Yes	Yes	Yes
3	23	Male	Left	3 times	No	No	Yes
4	37	Male	Left	12 times	Yes	No	Yes
5	26	Male	Left	15 times	No	Yes	Yes
6	6	Female	Left	twice	No	No	No
7	29	Female	Left	8 times	Yes	No	Yes
8	6	Female	Left	no	Yes	Yes	Yes
9	39	Female	Left	once	Yes	Yes	Yes
10	19	Female	Right	6 times	Yes	No	Yes

*IO* internal orifice, *PFA* pyriform fossa apex

### Diagnosis

Pre-operative routine examinations included fiberoptic laryngoscope and barium swallow X-ray to locate the internal orifice, and to evaluate patency of the internal orifice with cervical lesion. And cervical CT or MRI was performed to further evaluate the anomaly, its cervical extent, and its relationship with vital structures.

### Surgical protocol

Surgical management is undertaken in a 2-step fashion during a single operative procedure.

**Fig. 1:**
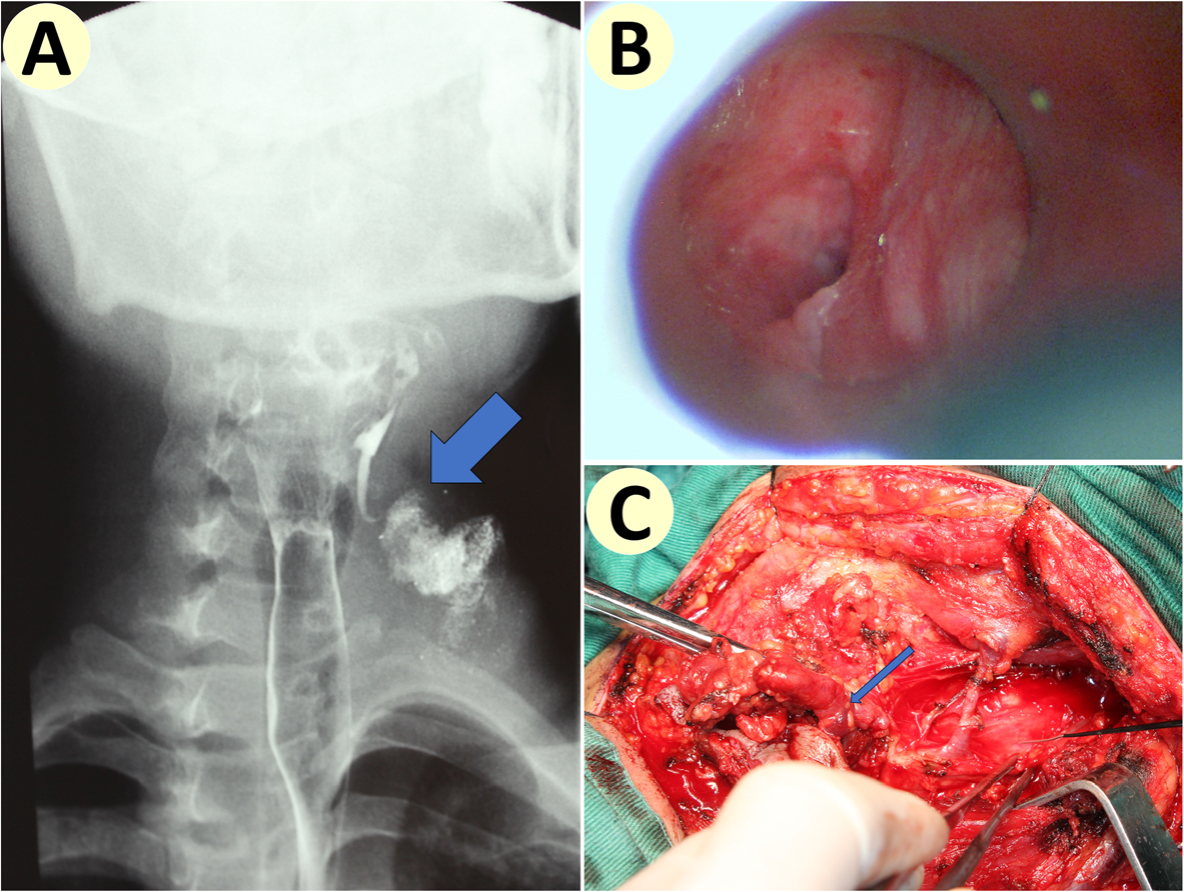
Pre-operative diagnosis and intra-operative confirmation. **a** Outflow of barium from pyriform sinus into the fistula tract and further into the massive cervical lesion could be seen on X-ray film (see blue arrow). **b** Intra-operative direct laryngoscope visualized the internal orifice at the apex of pyriform fossa of the same patient. **c** Intra-operative confirmation of entry into pyriform fossa at the inferior cornu of thyroid cartilage (see blue arrow) of the same patient

Firstly, direct laryngoscope was deployed to locate and confirm the internal orifice (Fig. 1). Then a blunt-tip suction tube was inserted, and methylene blue was injected into it. If external orifice still existed, methylene blue was also injected into it. If recent infection has occurred, we would delay surgery for 6 weeks in order to allow for inflammation and edema to resolve as this may impair demonstration of the tact [4].

Secondly, design of skin incisions depended on cervical scar. In general, the incision was made to contain all scar tissue and elongated bilaterally to allow sufficient flap elevation; if there was no cervical skin involvement, a classic step ladder incision was adopted (see Fig. 4). Then the anomaly and surrounding inflammatory tissue was dissected together, taking caution to protect the carotid sheath. If the anomaly was found to pass through thyroid gland, then RLN and SLN, especially its external branch, were dissected and protected. Intra-operative nerve monitoring was routinely used to ensure accurate identification and protection of RLN and SLN. Then the anomaly was traced internally to find its entry point into hypopharynx. Occasionally, the posterior part of the thyroid cartilage needed to be removed to gain better exposure, but at least 1 cm above the inferior cornu of the thyroid cartilage should be kept intact to avoid damage to RLN.

Now, direct laryngoscope would be deployed again, and a blunt-tip probe would be inserted through the internal orifice to help locate the connection between cervical lesion and hypopharynx in the open cervical surgical field, and we referred to this maneuver as “pharyngeal confirmation”. Then the attachment of the anomaly to the internal orifice at PFA was divided and ligated with purse-string sutures. Inferior pharyngeal constrictor would be used to strengthen the exposed hypopharyngeal wall. At the end of a procedure, the surgical field was irrigated with hydrogen peroxide, and saline. A high negative pressure drain was put in the surgical field, and neck incisions were closed with multi-layered interrupted sutures.

## Results

### Pre-operative identification of internal orifice

**Fig. 2:**
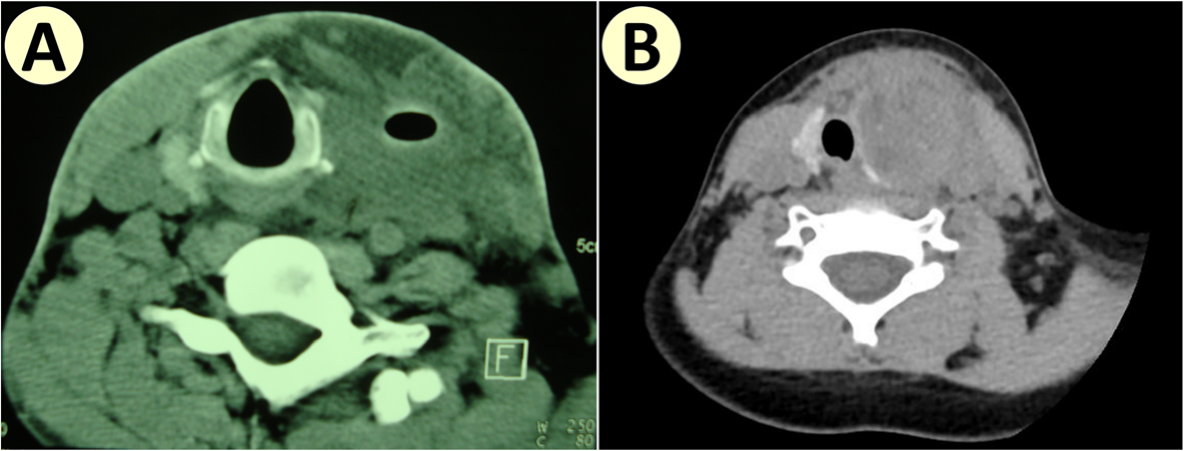
Close relationship between fourth branchial fistula and thyroid gland on CT scan. **a** Left-sided infected fourth branchial fistula, whose tract can be seen at the center of the inflamed mass. **b** Left-sided infected fistula, whose tract isn’t obvious

**Fig. 3:**
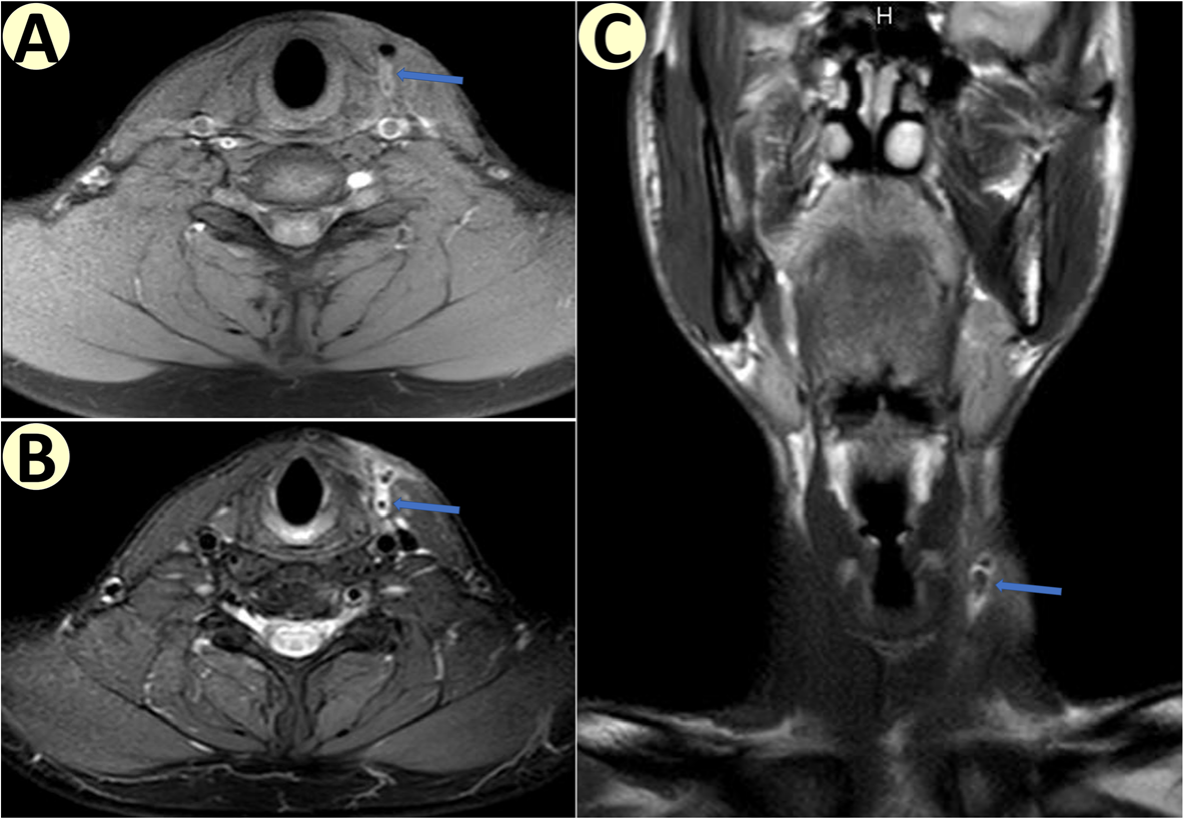
MRI of a left side fourth branchial fistula, as indicated by blue arrow. **a** T1WI axial image. **b** T2WI axial image. **c** T2WI coronal image

In 7 patients, visualization of internal orifices at PFA by fiberoptic laryngoscope confirmed diagnosis of fourth branchial apparatus anomalies. In 5 patients, outflow of barium from PFA could be observed on X-ray, indicating diagnosis of fourth branchial apparatus anomalies, and demonstrating patency between hypopharynx and cervical lesion (see Fig. 1). In total, 8 (80%) patients were diagnosed pre-operatively. In 4 patients, cervical CT or MRI revealed patent central fistula tracts within cervical lesion (see Figs. 2 and 3). Nine patients had previously presented with repeated neck infections and undergone incision and drainage.

### Treatment and intra-operative confirmation of internal orifice

Initial direct laryngoscope confirmed or identified PFA internal orifice in 9 (90%) patients. Then skin incision was made, and subplatysmal flap was elevated. Blue-walled fistula tracts could be seen penetrating the platysma or a blue-walled cyst was found under platysma (in the case of fourth branchial pouch sinus). Then dissection along the tract/cyst was undertaken, preserving the integrity of the wall to prevent pollution of the surgical field by the blue dye. Then the tract could be followed to the vicinity around carotid sheath, and careful dissection to separate the tract from carotid sheath and vagus nerve was crucial. Granulation tissue, scar tissue, or even adhesion patch had been found between the tracts and carotid sheaths, in cases with repeated episodes of neck infections. In 6 cases, the adhesion between the fistula tract and the thyroid gland was so severe that the superior part of a thyroid lobe was removed with the adhesion patch, to facilitate further dissection and to keep the integrity of the tract. The RLN was dissected and protected in all 10 cases.

In 3 cases, the tracts were followed to the inferior cornu of the thyroid cartilage and entered the cricothyroid space, and their fistulae were confirmed as fourth branchial fistula (see Fig. 1). While in 7 other cases the tracts and inflammation mass surrounding them were followed to the posterior aspects of the lower half of the thyroid cartilage, but the exact points of entry into hypopharynx could not be determined. Sharp dissection of the mass was undertaken until blue stained tract wall could be seen, and the entry points were all identified to be just superior or posterior to the inferior cornu, and these conditions were confirmed or identified as fourth branchial apparatus anomalies.

Now, direct laryngoscope was deployed again to perform pharyngeal confirmation through the internal orifice, for the 9 patients whose internal orifice had been found during the initial direct laryngoscopy. Then purse-string sutures were used for the final ligation of the tract, including its pyriform fossa attachment, to prevent future infection originating from hypopharynx.

In one case (patient No. 6), internal orifice wasn’t found pre-operatively, or intra-operatively, despite our best efforts, so only the cervical lesion with surrounding inflammatory tissues were resected.

Recovery of all 10 patients were uneventful. No vocal cord paralysis, or post-operative hematoma occurred. The drain was removed when daily drainage was less than 10 ml. After 7 days of fluid feeding, all patients returned to normal diet and didn’t require nasogastric tube feeding.

### Follow-up

Duration of follow-up was between 6 months and nearly 6 years (median length of 4 years). Thus far there have been only one recurrence. Recurrence was detected in a 6 years old girl (patient No. 6), at 1 year after surgery. At 6 weeks after relief of her cervical infection by surgical drainage and antibiotics, a second surgical exploration was scheduled. Her internal orifice was eventually found at PFA and properly managed. No recurrence has been detected after follow-up of nearly 2 years.

## Discussion

Branchial anomalies can present as a spectrum of conditions such as cysts, sinuses and fistulae. There diagnosis can be difficult and the clinical course may be prolonged before the correct diagnosis is made. This was reflected in our series where the majority of patients had undergone multiple previous unsuccessful treatment. A branchial anomaly should be considered in the differential diagnosis of a young patient presenting in the first or second decade of life with an infected neck sinus.

Clinical diagnosis involves identifying the orifice in the piriform fossa on laryngoscopy. This can be supported by contrast imaging, which can reveal contrast delineated sinus tract [17], or shallowing or even obliteration of pyriform fossa [18]. Better tissue contrast of MRI can provide the relationship of glandular tissue to the mass, and hence acts as a roadmap prior to surgery [17].

Surgical excision of third and fourth branchial apparatus anomalies is very challenging, due to its complex course, intimate relationship to various important structures, and high risk of recurrence. Some of our patients have undergone repeated unsuccessful surgical treatment, which made surgery more challenging due to scar tissue. A thorough understanding of the complex anatomy and potential course of the tract is very important.

For a third branchial fistula: from an external opening anterior to sternocleidomastoid muscle, the tract runs deep to platysma, along the carotid sheath, passes deep posterior to the internal carotid artery, between the glossopharyngeal nerve above and hypoglossal nerve below, through the thyrohyoid membrane, and enters the pharynx in the region of the pyriform fossa [3]. And for a fourth branchial fistula, it would begin at the pyriform fossa, exit the larynx near the cricothyroid joint, pass between the RLN and SLN, and then take a different course depending on whether it was on the right or left side of the neck; and the theoretical course after that was so convoluted that it had never be clinically observed [4].

**Fig. 4:**
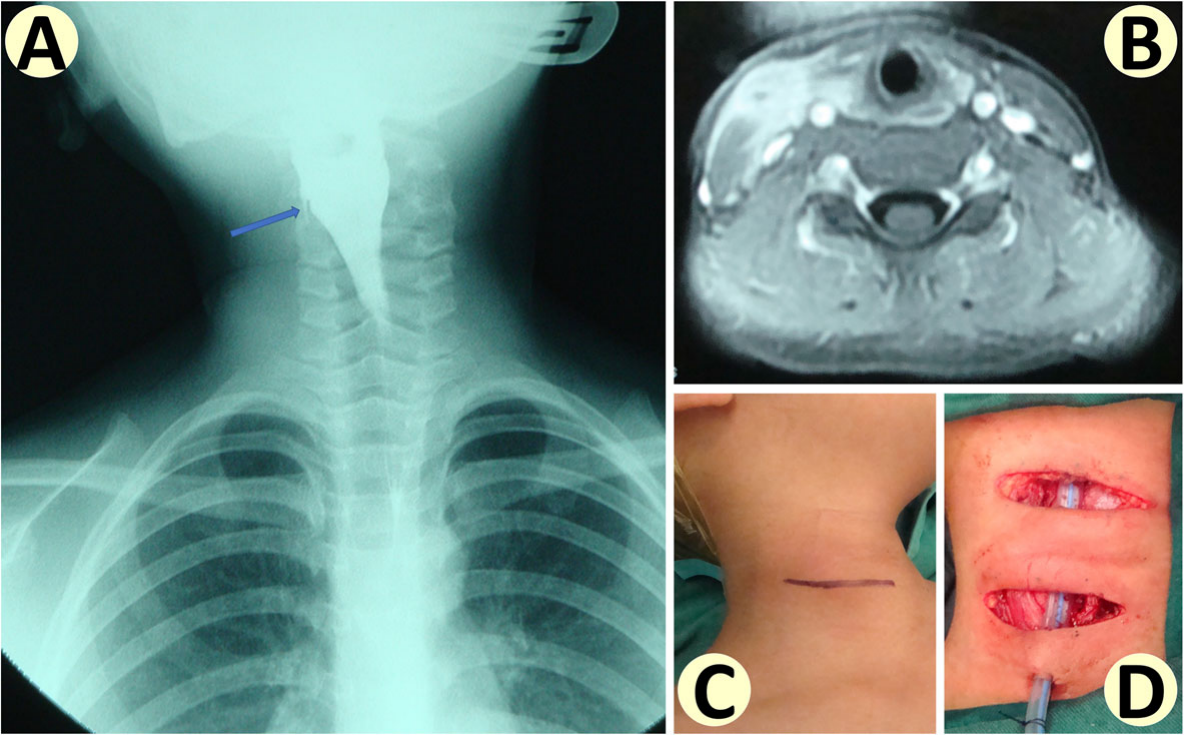
Right-sided fourth branchial pouch sinus. **a** Tiny amount of right-sided barium outflow from pyriform sinus can be seen on X-ray film (blue arrow). **b** Right-sided massive cervical inflammation of the same patient. **c** Pre-operative cervical photo shows no cervical cutaneous orifice. **d** Step ladder incision for this patient, with drainage tube in surgical field

Fourth branchial anomalies are very rare, and the largest case series reported so far was the 52 cases by Rossi M.E. in 2019, which was a multicentric retrospective review on cases collected over 19 years [19]. Verret D.J. et al. reported 10 cases in 2004 [10], Lu W.H. et al. reported 8 cases in 2012 [20], Pal I. et al. reported 7 cases in 2018 [21], Nicollas R. et al. reported 6 cases in 1998 [15], Arunachalam P. et al. reported 5 cases in 2015 [22], and Waston G.J. et al. reported 5 cases in 2013 [23], as shown in Table 2. Right-sided fourth branchial apparatus anomalies are extremely rare, which means we are very lucky to have encountered 2 cases (see Fig. 4). Rossi M.E. reported 3 right-sided cases [19], while Pal I. et al. reported 1 right-sided case [21].

**Table 2:** Reported case series of fourth branchial apparatus anomalies

Case series	Patient number	Treatment	Recurrence	Complication
This series	10	OPS	1 (10.0%)	None
Rossi M.E. et al	52	ECIO in 38	11/38 (28.9%)	None
		OPS in 14	3/14 (21.4%)	5/14 (35.7%)
Verret D.J. et al	10	ECIO	None	None
Lu W.H. et al	8	OPS	None	1/8 (12.5%)
Pal I. et al	7	OPS	None	None
Nicollas R. et al	6	OPS	None	2/6 (33.3%)
Arunachalam P. et al	5	ECIO in 4	None	None
		OPS in 1	None	None
Waston G.J. et al	5	ECIO	None	None

*OPS* open neck surgery, *ECIO* endoscopic cauterization of internal orifice

When third or fourth branchial apparatus anomalies were suspected, cervical exploration procedures should be performed by experienced surgeons. It is our experience that blue dying and identification of entire tracts, especially their entry points into the hypopharynx from inside the cervical surgical field, with pharyngeal confirmation through direct laryngoscope, was the key for complete resection and recurrence prevention of fourth branchial apparatus anomalies. Although some authors did not use blue dying of tracts in their procedures, especially those that only cauterize internal orifices [19, 24]; other authors advocate the use of blue dying in the identification of the filiform tract within the fibrous tissue to achieve complete excision, especially in open neck procedures [14, 25].

The only recurrence in this cohort, is the result of failed identification of the internal orifice. And intra-operative direct laryngoscope is the best method to locate the internal orifice, because pyriform fossa can be better exposed and examined under general anesthesia, as shown in our cohort to be effective in 9 (90%) cases. Effectiveness was reported to be 61.2% (70% in our series) for fiberoptic laryngoscope [25]. And effectiveness of barium swallow X-ray was reported to be 61.2% by Li Y. et al. [25], or 75.0% (6/8) by Lu W.H. et al. [20], while it is 50.0% (5/10) in our series.

Involvement of the thyroid gland can occur, and may be the underlying cause of repeated episodes of suppurative thyroiditis, as was observed in our cohort (see Figs. 2 and 3). And hemithyroidectomy may be needed to dissect the tract completely [7, 13]. Yet, we did not remove an entire thyroid lobe, we only remove the part of the thyroid lobe that was adherent to the fistula tract. In this way, the thyroid and parathyroid glands are better protected, and the residual thyroid lobe can be used to strengthen hypopharyngeal wall and isolate potential infection from carotid sheath. We routinely use intra-operative nerve monitoring to ensure accurate identification and protection of RLN and SLN (especially its external branch). In this case series, there has been no injury of RLN or SLN.

Although cauterization of the internal orifice with trichloroacetic acid, silver nitrate, plasma, electrical cautery, or laser has been recommended by many authors [10, 19, 21–23], it does have a risk of RLN or even esophageal injury, and is not a definitive treatment of this condition with significantly increased chance of recurrence [18, 19, 24]. In a recent Chinese cohort of 146 cases of pyriform fossa fistula from single institution, plasma cauterization of the internal orifice was the initial treatment. Nine (6.2%) patients experienced post-operative hoarseness. During follow-up, recurrence was detected in 30 (20.5%) cases [18]. Whereas in the case series of Rossi M.E. et al., after endoscopic cauterization of internal orifice, recurrence was detected in 11/38 (28.9%) cases [19].

It is also worth noticing that repeated procedures of endoscopic cauterization of internal orifice might be need in cases of recurrence, and might need open neck surgery for refractory cases [19, 25]. So it is very important to master the surgical skills and experience on this peculiar and rare clinical condition, for head and neck surgeons. Our unique experience that might add to literature regarding this surgical procedure includes the following: initial direct laryngoscope examination of hypopharynx after anesthesia to identify internal orifice, blue dying of the entire lesion (appropriate amount of methylene blue will be injected into both internal and external orifices) and removal of all blue dyed tissue, routine RLN monitoring which can also be used to identify SLN, and pharyngeal confirmation with a probe that is introduced through internal orifice to indicate the connection between cervical lesion and hypopharynx which will be ligated with purse-string sutures.

## Conclusions

Routine pre-operative examination should include fiberoptic laryngoscope and barium swallow X-ray, when there is suspicion of fourth branchial apparatus anomalies. Direct laryngoscopy under general anesthesia is a reliable method of diagnosis. Complete surgical excision, including their hypopharyngeal attachment, is the treatment of choice and key to prevent recurrence. Surgical procedures can be very challenging and should be performed by experienced surgical team.

## Data Availability

All data, models, and code generated or used during the study appear in the submitted article.

## Abbreviations

PFA: Pyriform fossa apex
RLN: Recurrent laryngeal nerve
SLN: Superior laryngeal nerve
